# A Parallel Attractor Finding Algorithm Based on Boolean Satisfiability for Genetic Regulatory Networks

**DOI:** 10.1371/journal.pone.0094258

**Published:** 2014-04-09

**Authors:** Wensheng Guo, Guowu Yang, Wei Wu, Lei He, Mingyu Sun

**Affiliations:** 1 School of Computer Science and Engineering, University of Electronic Science and Technology of China, Chengdu, Sichuan, China; 2 Electrical Engineering Department, University of California Los Angeles, Los Angeles, California, United States of America; 3 Institute of Liver Diseases, ShuGuang Hospital, Shanghai University of Traditional Chinese Medicine, Shanghai, China; 4 Department of Microbiology, Immunology & Molecular Genetics, David Geffen School of Medicine, University of California Los Angeles, Los Angeles, California, United States of America; University of Rome Tor Vergata, Italy

## Abstract

In biological systems, the dynamic analysis method has gained increasing attention in the past decade. The Boolean network is the most common model of a genetic regulatory network. The interactions of activation and inhibition in the genetic regulatory network are modeled as a set of functions of the Boolean network, while the state transitions in the Boolean network reflect the dynamic property of a genetic regulatory network. A difficult problem for state transition analysis is the finding of attractors. In this paper, we modeled the genetic regulatory network as a Boolean network and proposed a solving algorithm to tackle the attractor finding problem. In the proposed algorithm, we partitioned the Boolean network into several blocks consisting of the strongly connected components according to their gradients, and defined the connection between blocks as decision node. Based on the solutions calculated on the decision nodes and using a satisfiability solving algorithm, we identified the attractors in the state transition graph of each block. The proposed algorithm is benchmarked on a variety of genetic regulatory networks. Compared with existing algorithms, it achieved similar performance on small test cases, and outperformed it on larger and more complex ones, which happens to be the trend of the modern genetic regulatory network. Furthermore, while the existing satisfiability-based algorithms cannot be parallelized due to their inherent algorithm design, the proposed algorithm exhibits a good scalability on parallel computing architectures.

## Introduction

The majority of human diseases is complex and caused by a combination of genetic, environmental and lifestyle factors, including cancer, Alzheimer's disease, asthma, multiple sclerosis, osteoporosis, connective tissue diseases, kidney diseases, liver diseases, autoimmune diseases, etc. The high-throughput-high-content gene screen technology is a possible way to uncover genetic and genomic approaches. The research interests are gradually shifted from single-gene disorders to polygenic relationship. Since a large number of potential biological and clinical applications are identified to be a solvable problem using network-based approaches. A Genetic regulatory network (GRN) and its functional biology are important to be utilized for the identification of mechanisms of the complex disease and therapeutic targets [1], [2].

The GRN consists of a collection of molecular species and their interactions. To understand the dynamical properties of a GRN, it is necessary to compute its steady states, which is also known as attractors. The attractor has a practical implication: a cell type may correspond to an attractor. For instance, the GRN of T helper has 3 attractors, which correspond to the patterns of activation observed in normal Th0, Th1 and Th2 cells respectively [3]. A number of methods have been proposed to model the GRN [4]. In these models, the Boolean network is a simple and efficient logical model for the GRN. It utilizes two states to represent the gene states of the GRN [5]. At a particular moment, the state set of all nodes in the Boolean network is called a state of the network. The graph formed by all states of the network is called a State Transition Graph (STG). In an STG, a fixed point or a periodic cycle is defined as an attractor that is corresponding to a steady state of a GRN. The interesting attractor finding is, however, identified as a NP-hard problem [6], [7].

Algorithms of finding attractors have been extensively studied in the past decade [3], [8]–[12]. A few of these algorithms are available as released tools, such as Genetic Network Analyser [13], SQUAD [14], CellNetAnalyzer [15], Odefy [16], Jemena [17], etc. All these existing algorithms can be categorized into four groups. The simulation-based approach is proposed to find attractors by choosing several initial states heuristically and to simulate the activation and inhibition for each initial condition [8], [13], [15]–[17]. It is, however, difficult to cover all the attractors in a GRN because the initial states are randomly generated. The rest three categories of algorithms find attractors by formulating the original problem as follows: binary decision diagram (BDD) problem [3], [9], [10], [14], satisfiability (SAT) problem [11], and aggregation problem [12]. The BDD is a data structure for describing a Boolean function. In a BDD-based algorithm, all relations of activation and inhibition between genes are represented as reduced ordered binary decision diagram (ROBDD or in short BDD) [3], [9], [10], [14], [18]. It is then that the Boolean operations are computed based on the BDD. The size of the BDD is determined both by the Boolean function and by the order of variables. Therefore it is exponential to the order in the worst case and a state explosion could happen, which limits the BBD based algorithms to simple Boolean networks only [3], [9], [10]. SAT-based algorithms avoid this problem by solving a set of satisfiable constraints alternatively without searching throughout the entire state space. It often leads to more efficient search because of the automatic splitting heuristics and applying different splitting orderings on different branches SAT-based algorithms are tailored for finding attractors in a large-scale Boolean network using SAT-based bounded model checking [11], [19]. These algorithms unfold the transition relation for *N* iterative steps to form a propositional formula and solve it using a SAT solver. In each iterative step, a new variable is used to represent a state of a node in a Boolean network. The number of variables in the propositional formula is, however, *N* times of the number of nodes in the Boolean network, if a transition relation is unfolded for *N* steps. Therefore the larger the number of nodes and unfolding steps are, the higher the computation complexity will be. An aggregation algorithm is also proposed to find the attractors in a large-scale Boolean network [12]. The min-cut aggregation [20] and max-modularity aggregation [21] can be utilized to partition the Boolean network. In each subnetwork, the Johnson’s algorithm [22], [23] and semi-tensor product approach [24] can be applied to find attractors, whereas, the aggregation algorithm only provides a framework without an efficient implementation.

To tackle the aforementioned problems, we are proposing an algorithm that partitions a Boolean network into smaller blocks, such that SAT algorithm can be applied efficiently on these smaller blocks for finding attractors. Furthermore, the proposed algorithm can be parallelized and better performance is exhibited on a multicore architecture. The proposed algorithm is tested using two set of benchmarks, test cases acquired from literature [8], [25]-[30], which are typically very small, and larger test cases generated in an R environment [31] based on the BoolNet package [32]. On the smaller cases, the runtime of the proposed algorithm is comparable to the state-of-the-art solver BNS [11]. However, on the larger test cases, which are the trend of the modern genetic regulatory network, the proposed algorithm outperforms BNS.

The rest of this paper is organized as follows. The model, definitions, and algorithm description are provided in Section 2. The experimental results and discussion are illustrated in Section 3. Finally, Section 4 concludes the paper.

## Methods

### The Boolean Network Model

A Boolean network can be considered as a directed graph 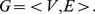 Each node 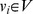 has an associated state variable 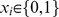 and a state transition function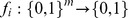, where 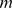 is the number of nodes related to node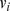. The edge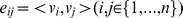 directing from node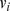 to node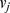 describes that the next state of node 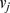 depends on the current state of node 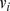.

At the time step *i*, the state of the Boolean network is a binary vector 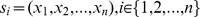. If the states in 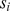 are updated simultaneously, the Boolean network is called a synchronous Boolean network (SBN). When only one state variable, 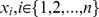, is updated at each time step, it is called an asynchronous Boolean network (ABN). In the SBN, each state vector has a unique next state in STG. All states eventually converge to an attractor. The SBN is used to model a GRN in the following discussions.

In a Boolean network, the transition relation, 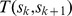, can be represented by the following formula:
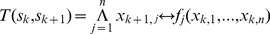
(1)where 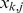 is the state variable of node 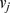 at the time step *k*, 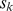 and 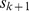 stand for states of the Boolean network at the time step *k* and *k+1* [19]. Considering an STG corresponding to this SBN, 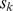 and 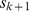 are the source and the destination node of one edge. Therefore, a path of the STG can be defined as the following expression:



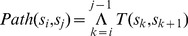
(2)In the SBN, the next state of a state in an STG is unique. Hence, the next state of a state in an attractor must be in the attractor. According to the definition of an attractor and expression of the path, we have the following theorem1:

***Theorem 1:*** In the STG of an SBN, if a path includes an attractor, the last state of the path must be in the attractor.

*Proof:* In the STG of an SBN, if a state is in an attractor, its next state must be in the attractor. Suppose the last state of the path is not in the attractor, then the state before the last state in this path cannot be in the attractor. Therefore, no state in the path is in the attractor. It is contradictory with the statement that the path includes an attractor.

**Figure 1:**
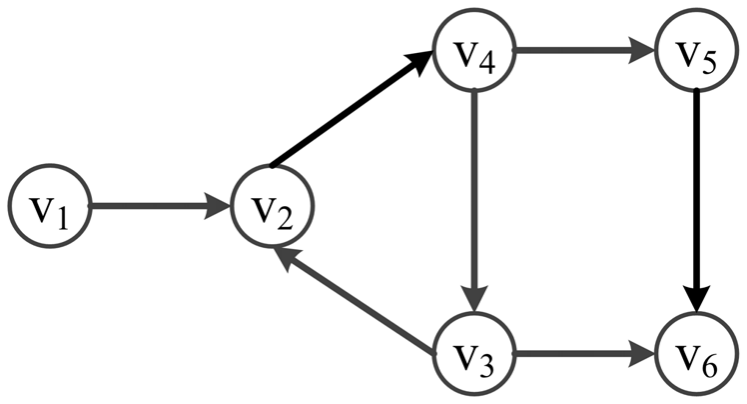
A 6-node Boolean Network. It is a general model of a GRN. A node/describes a gene in the GRN. A directed edge/expresses the interaction of activation and inhibition between two genes. The next state of a node/is a Boolean function of the previous states of the nodes which are predecessors of v_i_.

A Boolean network with six nodes is illustrated in Figure 1 as an example. It is a general model of a GRN, where the node 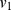 is an initial node. The value of state variables at next time step will be computed based on the following transition functions:
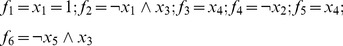
(3)


**Figure 2:**
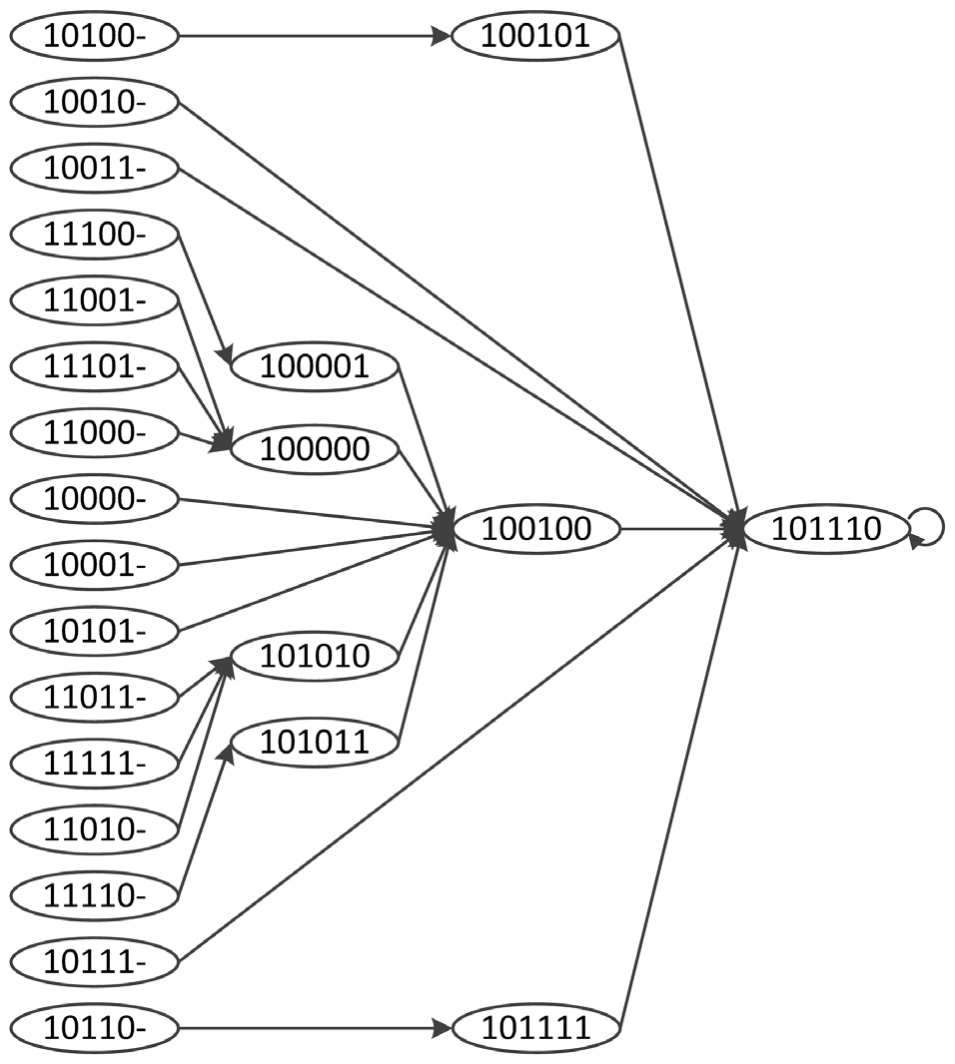
The State Transition Graph of Boolean Network. Each state is a 6-tuple 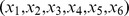. A directed edge indicates the state transition.

The corresponding STG is shown in Figure 2. The initial value of the state variable 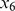 does not affect the next state of any node. Therefore, in the first column, we denote the initial state of 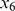 as “−”. In the last column, the state “101110” is an attractor. All states eventually arrive at that attractor. If a path includes “101110”, for example, “
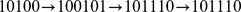
”, the last state of the path must be “101110”. In other words, the next state node of the state node in the attractor must be in the attractor.

### The Boolean Network Partition and Gradient Calculation

To decrease the computation complexity, we partition the Boolean network into several blocks based on the coupling between nodes in Boolean network. Then we find the attractors by computing the state transition of each block. Below, we present the four definitions for the Boolean network partition and gradient calculation.

***Definition 1:*** **Strongly Connected Component (SCC)** is a maximal strongly connected subgraph in a directed graph, 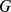. Here a subgraph 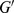 is strongly connected if there is a path from each node to every other node in 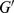.

In particular, the graph 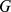 turns out to be a directed acyclic graph (DAG) if we consider all its SCCs as super nodes. In the DAG, we define the node without incoming node as root node, and the node without an outgoing edge as leaf node.

***Definition 2:*** **Gradient** of a node is the length of the longest path from any root node to the node.

***Definition 3:*** **Block** is a set of nodes with continuous gradient. The graph can be considered as a single block or partitioned into multiple blocks without overlap.

***Definition 4:*** **Decision node** of a block is a node that has an edge pointing to any node in this block. It is named decision node because the value of its state variable determines the state of the block.

Obviously, in a DAG, the state of a block is determined by all its decision nodes of the block.

**Figure 3:**
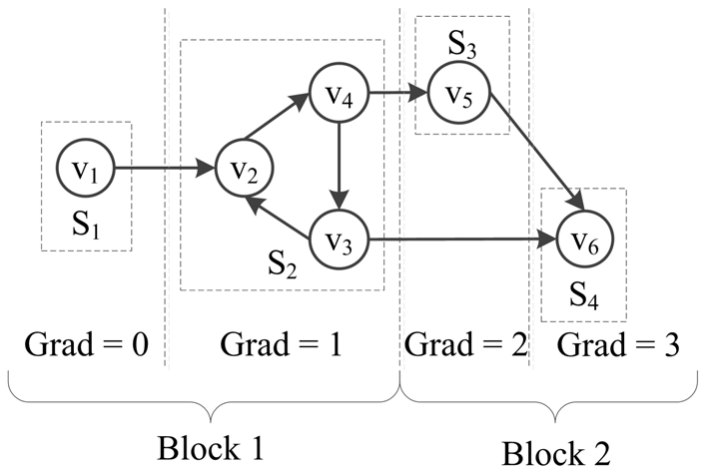
The SCCs, Gradients and Blocks of the Boolean Network.

A Boolean network with 4 SCCs, 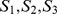, and 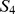, are illustrated in Figure 3. Considering each SCC as an abstract node, called a supernode, the Boolean network can be mapped to a DAG, where 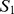 is a root supernode and 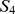
is a leaf supernode. Assuming the gradient of 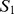 is 0, then the gradients of other SCCs are Grad(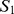) = 0, Grad(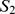) = 1, Grad(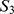) = 2, Grad(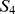) = 3 accordingly. This network can be partitioned into two blocks, 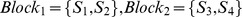. The decision nodes of these blocks are 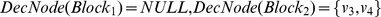, respectively.

### Algorithm

According to the network partition discussed in the previous section, we can find the attractors of a Boolean network by finding the attractors of each block. An attractor in a block is called a local attractor to distinguish the attractor in the entire Boolean Network.

An attractor can be expanded to the following pathway form based on expression (1) and (2):
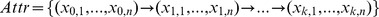
(4)where 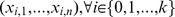 represents a state in the attractor, and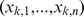 is the state after 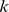 time steps from 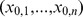. When a Boolean network is partitioned into blocks, the state vector 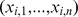 is also divided to multiple parts. Hence, an attractor of Boolean network is a combination of local attractors of all blocks.

First, we find local attractors of the block including the root SCC and get the solution sequences of all local attractors. Second, local attractors of the neighbor block are computed based on the solution sequence of each decision node. We combine the solutions in the first two steps to form a new solution set. The solutions can be computed step by step until all the blocks are computed.

Because a block has less constraint than the entire Boolean network, the total number of local attractors in a block is greater than or equal to the number of attractors in the Boolean network. To decrease the number of redundant solutions in a block, we need to consider the coupling between SCCs. In the meantime, the computation complexity increases along with its block size. Therefore we can construct a block according to the following steps: 1) get the lowest gradient of SCCs that are not in any block as the initial gradient of a new block; 2) search for the SCC with the highest gradient among all SCCs that are directly connected to the initial gradient SCCs, and configure the highest gradient as the maximum gradient of the new block; and 3) form a block with all the SCCs whose gradients are from the initial gradient to the maximum gradient.

A solution sequence of decision nodes is required to find the local attractors of a neighbor block. There could be three different kinds of solution conditions while finding the local attractors: 1) only one solution and this solution will be combined to the previous solution; 2) no solution, and previous solution will be deleted; and 3) two or more solutions, while each solution forms a new solution together with the previous solution.

**Figure 4:**
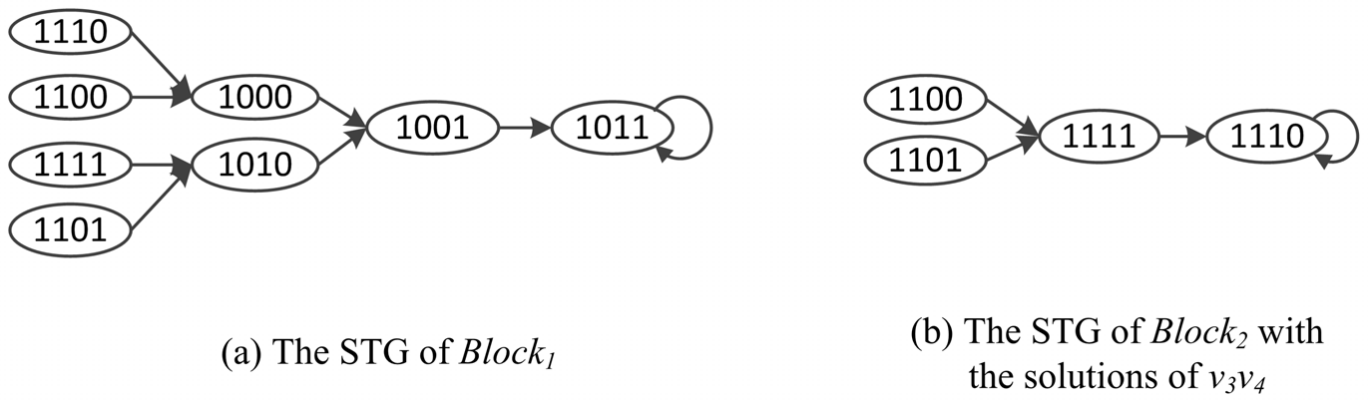
The STG of Blocks in Boolean network.

In Figure 3, we partition the Boolean network into two blocks, 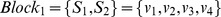
and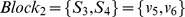. According to the transition function (3), the STGs of 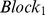 and 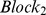 are illustrated in Figure 4. In Figure 4(a), the state variable sequence is 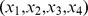. We get a local attractor 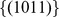 for 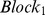. In the meantime, we get the solution sequence 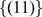 for the decision nodes 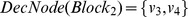. That means nodes 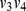 will repeatedly be set to the sequence 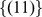 when block 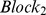 is computed. Then the STG of 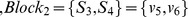
with decision nodes are presented in Figure 4(b), where the state variable sequence is 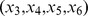. Thus, we find the local attractor 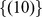 of 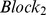. Eventually, we can get the attractor 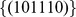 as a combination of 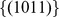 and 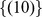.

Comparing Figure 2 and Figure 4, the state space is decreased drastically. As a consequence, our algorithm need less runtime to find the attractors because of the network partition.

### Implementations of the Algorithm

#### Sequential implementation of the algorithm

The pseudocode of the sequential version of the proposed algorithm implementation is presented in Algorithm 1. The Gabow’s algorithm [33] is adopted to compute SCCs. Then the gradients of SCCs are computed. The *startGrad* and *endGrad* describe the initial gradient and the maximum gradient of SCCs in a block respectively. To determine a block, we use *getMaxGrad* function to get the maximum gradient of SCCs which will be included in the block. To find the local attractors of a block, the SAT-solver that is implemented based on MiniSat [34] and it is used to solve the paths of a particular length *N* in the STG of the block. The STG of the block is created based on the solution sequence of the decision nodes and transition relation of the block. The *extend* function is designed to extend the solution sequence of decision nodes into paths of the STG of a block. A satisfying assignment solved by the SAT-solver is corresponding to a valid path in the STG of a block. Based on Theorem 1, we can determine whether the solution includes a local attractor. If the solution does not include a local attractor, it means the path is too short to enter a local attractor. Thus, we increase the length of the path until a local attractor is found. The *assemble* function is developed to combine the local attractors to the attractor of the entire Boolean network. If there is no satisfying assignment, the computation of local attractors in the current block is done and the current basic solution is deleted. The aforementioned procedures are repeated until all blocks are traversed.

***Algorithm 1:* A sequential version of the algorithm for finding attractors in a Boolean network**

//Initialization.

1 *startGrad = 0;*//It is starting gradient of SCC in a block.

2 *res = NULL;*//It stores the set of solutions.

3 *curResNum = 0;*//It denotes the index of the current solution.

4 *resCount = 0;*//It stores the number of solutions.

5 *countOfDecisionNodes = 0;*//It records the number of decision nodes.

6 *decisionNodesSolSeq = NULL;*//The assignment sequence that has been solved from last level gradient SCCs.

//Identifying the SCCs and Gradient.

7 *SCCs = getSCC(G).*

8 *setGrad(SCCs).*

//Finding attractors.

9 *endGrad = getMaxGrad(startGrad);*

10 *while (startGrad< = endGrad){*

11   *While(resCount =  = 0 || curResNum<resCount) {.*

12    *F_0_ = getCNF(startGrad, endGrad);*

13   *decisionNodesSolSeq = getDecisionNodesSol(Res,startGrad, endGrad);*

14   *N = getCountOfNodes(F_0_) +sizeof(decisionNodesSolSeq);*//The *N* is the count of nodes in the problem.

15   *F_n_ = transNStep(F_0_, N);*//The *F_n_* is the state transition set by *N* time steps based on the transition relation F_0_.

16   *F_n_ = F_n_* 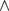 *extend(decisionNodesSolSeq, N);*

17   *while (SAT(F_n_)) {*

18    *If (isAttractor(satRes)) {/*/based on Theorem 1.

19     *assemble(Res[curResNum], satRes);*

20     *F_n_ = F_n_* 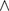 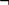 *satRes.*

21    *If (initGrad =  = 0) resCount^++^, curResNum^++^;*

22   *} else {*

23    *delete(Res[curResNum]);*

24    *F_n_ = transNStep(F_n_, N);*

25    N = N * 2;

26   *}*

27   *}*

28  *startGrad = endGrad +1;/*/set the starting gradient of the next block.

29  *endGrad = getMaxGrad(startGrad);*

30 *}*

31 *Print(Res);*//print all solutions.

#### Parallelization

Furthermore, conventional attractor finding algorithms based on SAT cannot be parallelized due to their inherent algorithm design. In this work, we take the parallelization into consideration during the algorithm design phase. As we partition the GRN into blocks and set gradients of the blocks, it is possible to map the SAT solving of different blocks to parallel hardware. In particular, if the first block of a Boolean network consists of multiple local attractors, our algorithm can fork a series of sub-processes to find attractors using the attractors found in the first block. The parallel version of the algorithm is described in Algorithm 2 briefly.

***Algorithm 2:* A parallel implementation of the algorithm for finding attractors in a Boolean network**

//Initialization and solving the first block.

1 *resFirst = getFirstBlockResult();/*/using the algorithm1 to find the attractors in the first block.

2 *base_res[1.CPU_NUM] = dispatch(resFirst, CPU_NUM);*//The resFirst is divided to CPU_NUM parts.

//Creating sub-process and solving attractors of the rest blocks.

3 *chPID = fork(CPU_NUM);*//Creating CPU_NUM sub-processes.

4 *if (chPID =  = 0){*//sub-process body.

5 *Result = Solve(base_res[cpu_index]);*//Solving the rest blocks based on the algorithm1.

6 *} else wait(subProcess);*//Parent Process wait until the sub-processes are over.

7 *Print(Result);*//print all solutions.

As the gradient identification guarantees unidirectional search of solving between the blocks and the independence between the local attractors of each block, our algorithm can be implemented in even more sophisticated parallel way than algorithm 2. After the solver finds a local attractor of the first block, a sub-process can be created to find local attractors of the other blocks. Similarly, our algorithm creates a series of sub-processes that are proportional to the number of attractors in Boolean network.

## Results and Discussion

We use the real GRN models in [8], [25]–[30] and N-K random Boolean networks [5], [35] as benchmarks. As the objective of the experiment is to find all attractors of a GRN, the proposed solvers, including both the sequential version and the parallelized version, are compared with BDD-based solvers, genYsis [3] and BooleNet [9] and the SAT-based solver BNS [11] in the experiment.

- genYsis [3]: the solver in SQUAD for finding all attractors [14]. It has three run modes: synchronous, asynchronous and synchronous-asynchronous combined. In our experiment, the genYsis is configured in the synchronous mode.
- Boolenet [9] and BNS [11]: the state-of-the-art algorithms based on BDD and SAT respectively.
- Proposed ST: the sequential version of the proposed algorithm.
- Proposed MT: the parallel version of the proposed algorithm.

Other tools, such as CellNetAnalyzer, Odefy and Jemena are not chosen because they are mostly based on simulation approach that cannot find all attractors [8], [13], [15]–[17]. All tests are performed on a machine equipped with Intel Xeon CPU @3.3 GHz 8-Core with 128 GB memory running Ubuntu 12.04.

### Comparison between SAT-based Algorithms and BDD-based Algorithms

We compared the BDD-based algorithms, genYsis, BooleNet with SAT-based algorithms, BNS and proposed ST. The runtime of these sequential algorithms on finding all attractors in real GRN models are shown in Table 1.

**Table 1:** The real Boolean network models of GRN.

Name	Number of nodes	Number * lengthof attractors	genYsis(sec)	BooleNet Realtime (sec)	BNS Realtime (sec)	Proposed ST Real time (sec)
Arabidopsis Thaliana	15	10*1	N/A	0.026	0.005	0.007
Budding yeast	12	7*1	0.142	0.051	0.012	0.013
Drosophilamelanogaster	52	7*1	N/A	>1000	0.057	0.093
Fission yeast	10	13*1	0.077	0.021	0.005	0.005
Mammalian cell	10	1*1,1*7	0.053	0.023	0.004	0.007
T-helper cell	23	3*1	0.085	0.059	0.005	0.006
T-cell receptor	40	8*1,1*6	0.826	0.047	0.011	0.017

Note: N/A denotes that the genYsis could not be executed in synchronous mode with experimental data in which the gene has a constant value with ‘HIGH’.

The results indicate that the SAT-based solvers are faster than BDD-based solvers in overall. In addition, the GRN models in Table 1 are all small and each runtime is relatively short. And our algorithm needs to compute strongly connected components and their gradient before solving attractors. Due to the overhead, the approach of the partition in the small GRN has not improved the performance of solving.

### Solver Runtime in Large-scale Random GRNs

**Figure 5:**
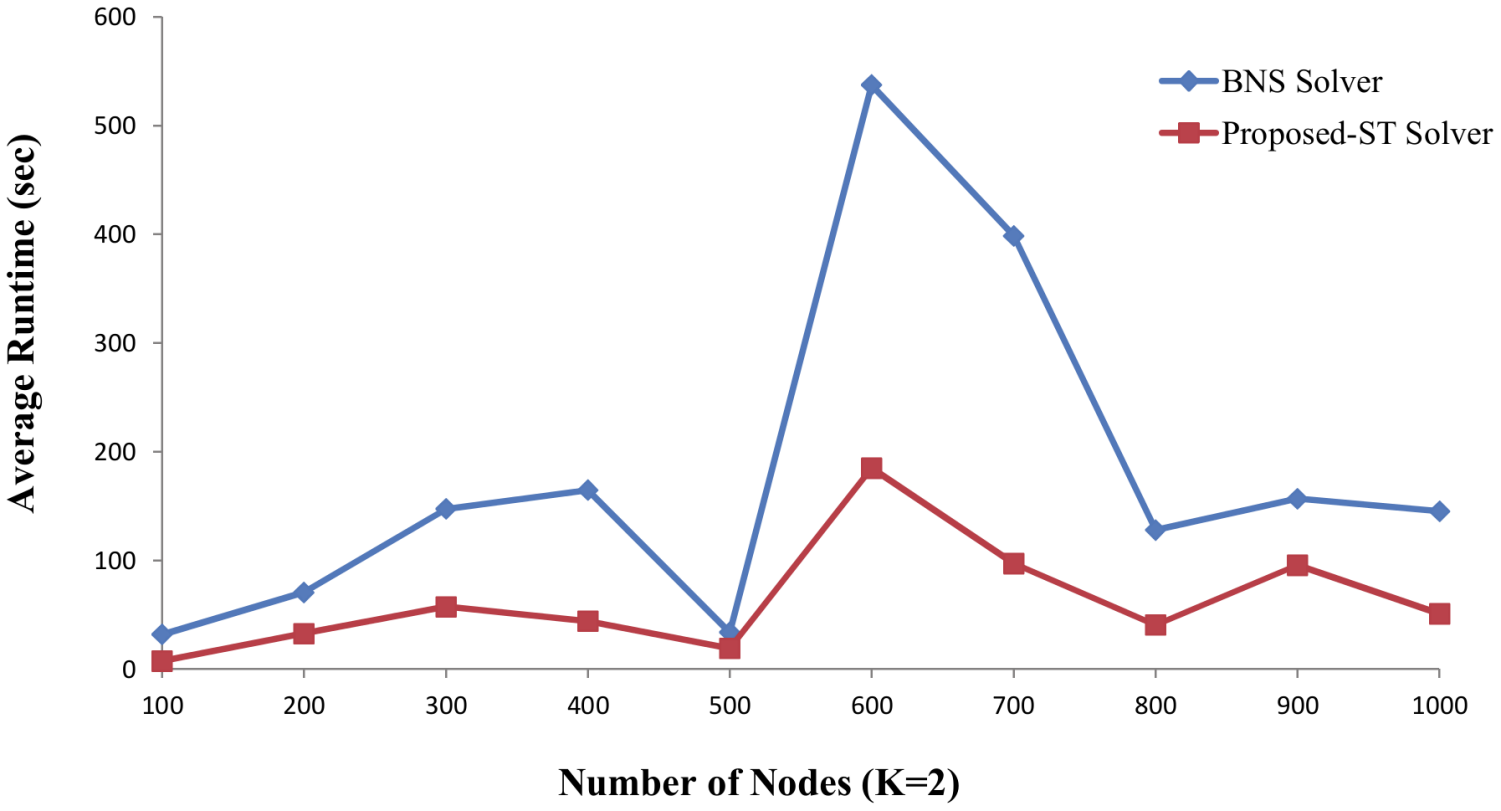
The results for finding attractor of randomly generated GRNs (K = 2). The parameters of *generateRandomNkNetwork* function are set to K = 2 and topology = “scale_free”. The number of nodes is from 100 to 1000. Five random instances are generated based on each number of nodes. The x-axis indicates the number of nodes. The y-axis is the average runtime of the five random instances corresponding to each number of nodes.

**Figure 6:**
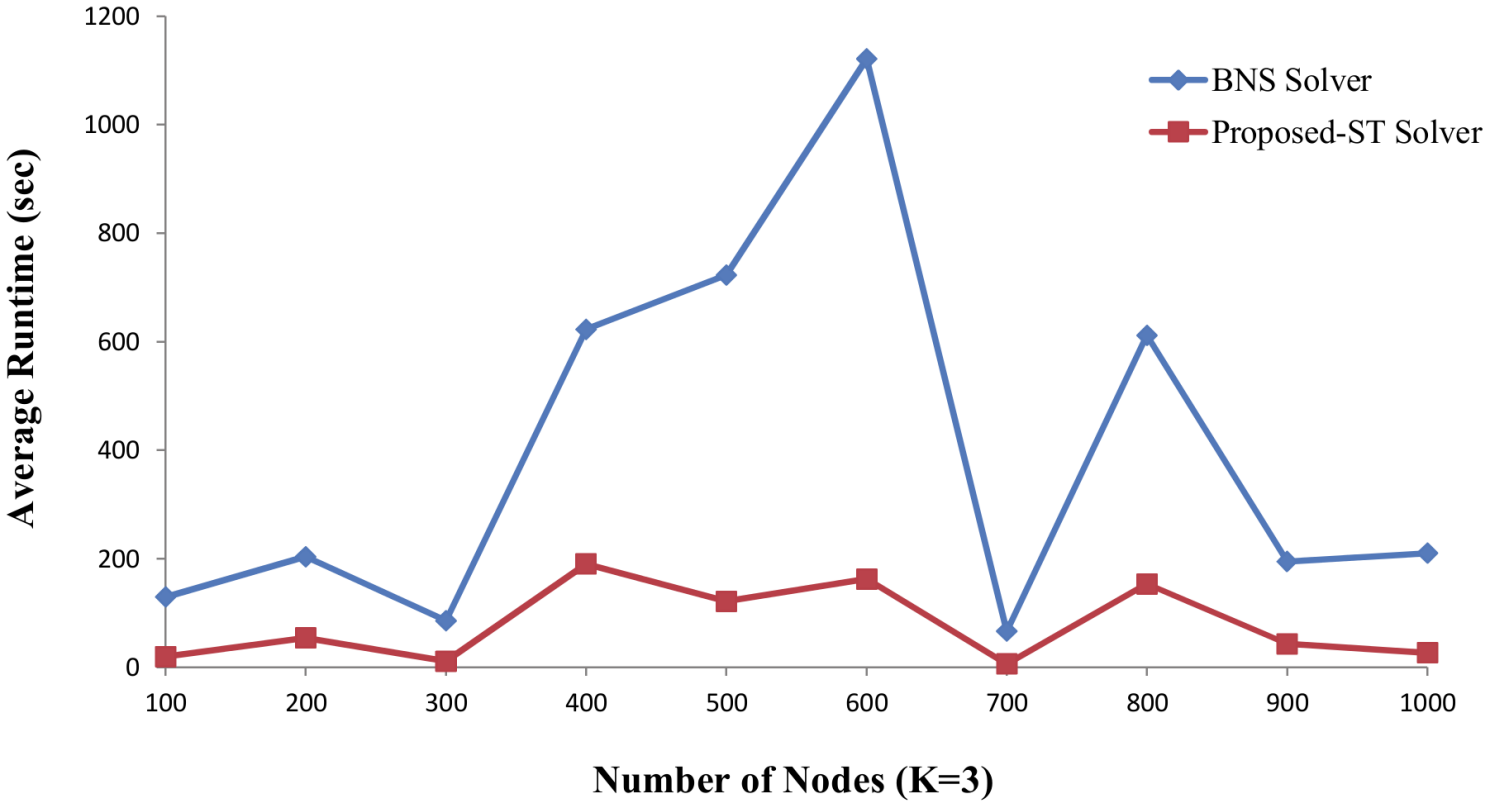
The results for finding attractor of randomly generated GRNs (K = 3). The parameters of *generateRandomNkNetwork* function are set to K = 3 and topology = “scale_free”. The number of nodes is from 100 to 1000. Five random instances are generated based on each number of nodes. The x-axis indicates the number of nodes. The y-axis is the average runtime of the five random instances corresponding to each number of nodes.

For human beings, the potential complexity of the resulting network is daunting. The number of functionally relevant interactions between the components of this network, representing the links of the interaction, is expected to be much larger. To test the performance of these algorithms on larger examples, we use the *BoolNet* package [32] in the *R* environment [31] to generate the N-K random Boolean networks. The parameters of *generateRandomNKNetwork* function are set to *K = 2* and *K = 3*, and *topology = “scale_free”* based on the literature [5], [35]. We generate a series of GRNs with the nodes from 100 to 1000 and choose 100 instances with a special number of nodes and parameter *K* in which the attractors can be found in limited time by BNS solver. The BNS solver and proposed ST solver run on the single core. The average runtime of test cases is showed in Figure 5 and Figure 6.

In Figure 5, the parameter *K* is set to 2 and 3 is set in Figure 6. The x-axis indicates the number of nodes and corresponding average runtime of five instances with same node number and *K* is on the y-axis. The results show the proposed ST has remarkably improvement in the large scale instances than the BNS solver. For example, in Figure 5, the average runtime of the five instances with node number 600 and *K* = 2 is 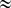 537 seconds in the BNS solver, 185 seconds in the proposed ST solver. In Figure 6, the average runtime with node number 600 and *K* = 3 is 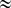
1121 seconds in the BNS solver, 162 seconds in the proposed ST solver. The higher time complexity is, the larger reduced time is.

**Figure 7:**
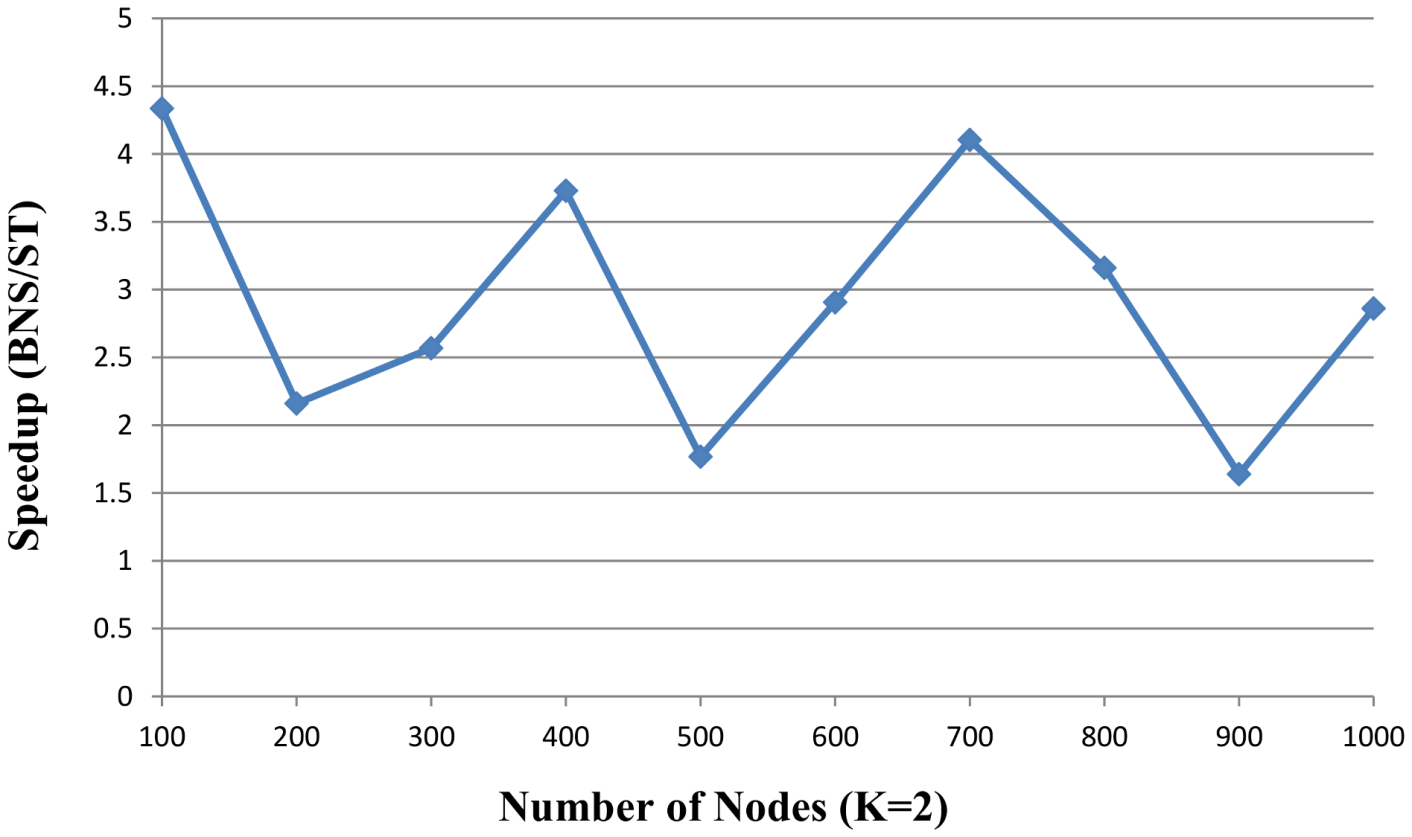
The speedup of proposed ST solver vs. BNS solver over the random instances (K = 2). The number of nodes is from 100 to 1000. Five random instances are generated based on each number of nodes. The x-axis indicates the number of nodes. The speedup on the y-axis is the ratio of BNS solver to proposed ST solver.

**Figure 8:**
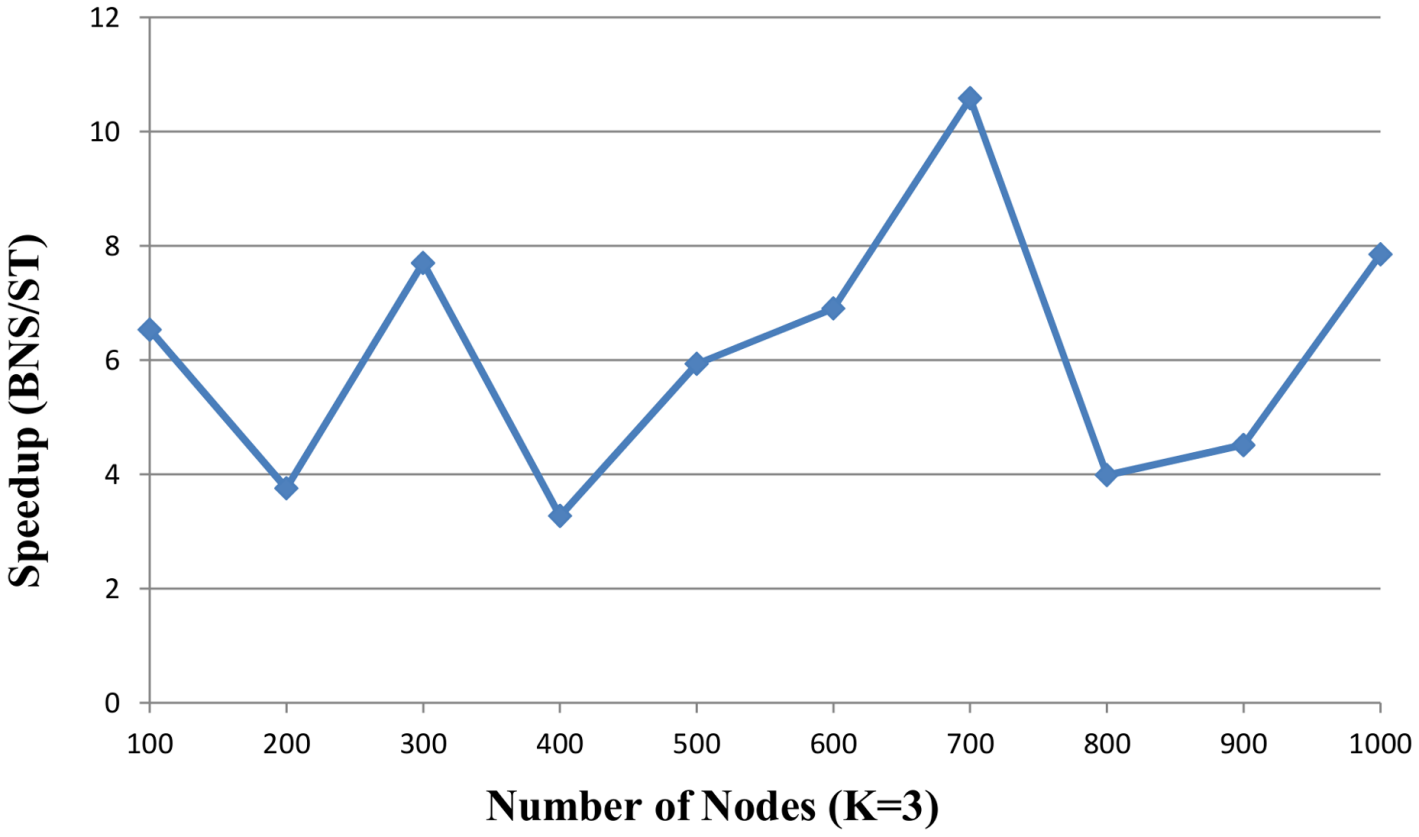
The speedup of proposed ST solver vs. BNS solver over the random instances with (K = 3). The number of nodes is from 100 to 1000. Five random instances are generated based on each number of nodes. The x-axis indicates the number of nodes. The speedup on the y-axis is the ratio of BNS solver to proposed ST solver.

Figure 7 and Figure 8 described the speedup ratios of random instances with *K* = 2 and *K* = 3, and the x-axis indicates the number of nodes and the speedup ratio is on the y-axis.

As we can see, the proposed algorithm is more efficient than BNS in large and complex random instances. The proposed ST solver is faster than the BNS solver which speedup ratios are 1.64–10.58 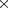 faster in the random instances. For example, in Figure 7, the speedup ratio of the sample with 900 nodes and *K* = 2 is 1.64 and the speedup ratio with 700 nodes and *K* = 3 is 10.58 in Figure 8. Compared to the BNS solver, the proposed-ST solver is 4.5 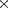 faster on average.

### Analysis of Parallelization of the Algorithm

**Figure 9:**
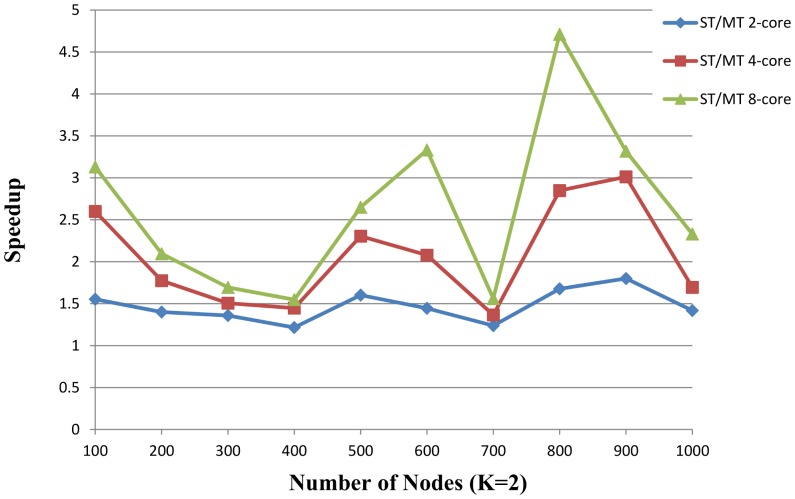
The speedup of proposed MT solver vs. ST solver over the random instances (K = 2). The proposed ST solver runs on a single core. The proposed MT solver runs on 2-core, 4-core and 8-core respectively. The number of nodes is from 100 to 1000. Five random instances are generated based on each number of nodes. The x-axis indicates the number of nodes. The speedup on the y-axis is the ratio of proposed ST solver to proposed MT solver.

**Figure 10:**
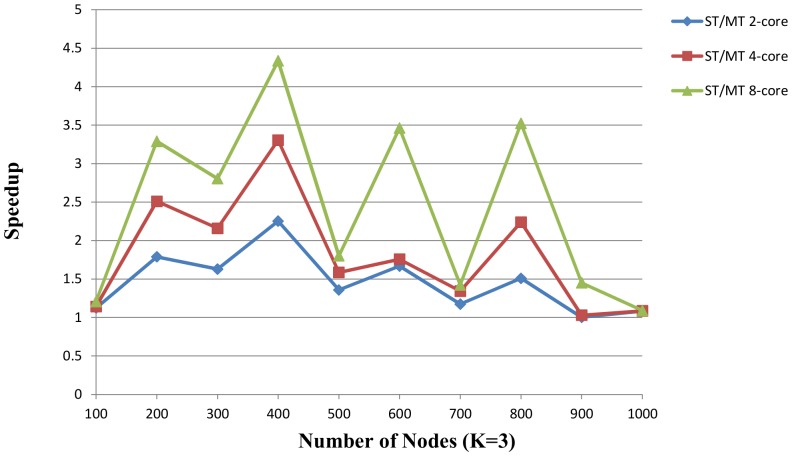
The speedup of proposed MT solver vs. ST solver over the random instances (K = 3). The proposed ST solver runs on a single core. The proposed MT solver runs on 2-core, 4-core and 8-core respectively. The number of nodes is from 100 to 1000. Five random instances are generated based on each number of nodes. The x-axis indicates the number of nodes. The speedup on the y-axis is the ratio of proposed ST solver to proposed MT solver.

The proposed MT takes advantage of the multicore to improve the performance of the proposed algorithm, while other SAT-based algorithm cannot. In the proposed algorithm, the network is partitioned into blocks and multiple sub-processes are created after the solution of the first block is computed. The total runtime after parallelization could be considered as:
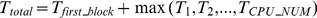
(5)where 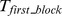 is the runtime for solving local attractors of the first block. 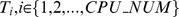 is the runtime of the *i* ^th^ sub-process based on the local attractors of the first block. *CPU_NUM* is the number of available concurrent cores on which the sub-processes can be executed. The minimum runtime of the proposed MT will be greater than the 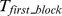. As a result, the scalability of parallel algorithm is only constrained by 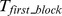. To further reduce the 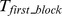, the first block of the proposed MT algorithm only includes the nodes with grad 0. In the meantime, we verify the scalability of the improved MT algorithm using the same test cases with Section 3.2, with 2, 4, and 8 concurrent cores. The results are illustrated in Figure 9 and Figure 10.

The results show a significantly improved performance compared with the sequential algorithm in 17 of 20 instances. In Figure 9, the average speedup is 1.47, 2.06 and 2.64 on 2-core, 4-core and 8-core. In Figure 10, the average speedup is 1.46, 1.82 and 2.44 on 2-core, 4-core and 8-core. Because the time of SAT solving is nonlinear, the speedup is not proportional to the number of cores. The performance of parallel algorithm is impacted by 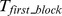 and the time of SAT solving could not be forecasted, therefore, the three instances (nodes 100, 1000, 900) have almost at the same runtime with sequential algorithm in Figure 10.

## Conclusion

In this paper, we presented an algorithm based on the partition and SAT for finding the attractors in a GRN modeled by the SBN. The algorithm uses the SCC and gradient to determine blocks and finds attractors in blocks based on the unfolding of the transition relation. We have verified the feasibility and efficiency of the algorithm by performing experiments on both small and large test cases. Our algorithm exhibits higher efficiency compared with other state of the art solvers (including BooleNet solver, BNS solver, and genYsis solver from SQUAD) in the larger and more complex cases, which would be the typical condition in real biological process model.

A potential future work could be studying the property of a GRN to realize the adaptive size of block to improve the performance of the algorithm, since the performance of solvers is also related to the structure of the network, and it is not proportional to the number of nodes.
